# Enterovirus D68 serosurvey: evidence for endemic circulation in the Netherlands, 2006 to 2016

**DOI:** 10.2807/1560-7917.ES.2019.24.35.1800671

**Published:** 2019-08-29

**Authors:** Eveliina Karelehto, Gerrit Koen, Kimberley Benschop, Fiona van der Klis, Dasja Pajkrt, Katja Wolthers

**Affiliations:** 1 Department of Medical Microbiology, Laboratory of Clinical Virology, Amsterdam University Medical Center, University of Amsterdam, Amsterdam, the Netherlands; 2 National Institute for Public Health and the Environment, Bilthoven, the Netherlands; 3 Department of Pediatric Infectious Diseases, Emma Children’s Hospital, University Medical Centers, University of Amsterdam, Amsterdam, the Netherlands

**Keywords:** enterovirus D-68, EV-D68, picornavirus, non-polio enteroviruses, acute flaccid myelitis, AFM, outbreak, surveillance

## Abstract

**Background:**

Enterovirus D68 (EV-D68) has caused major outbreaks of severe respiratory illness worldwide since 2010.

**Aim:**

Our aim was to evaluate EV-D68 circulation in the Netherlands by conducting a serosurvey of EV-D68 neutralising antibodies (nAb) among the Dutch general population.

**Methods:**

We screened 280 sera from children and adults in the Netherlands and used two independent sets of samples collected in the years 2006 and 2007 and in the years 2015 and 2016, time points before and after the first EV-D68 upsurge in 2010. Neutralisation capacity of the sera was tested against the prototype Fermon EV-D68 strain isolated in 1962 and against a recent EV-D68 strain (genotype B3) isolated in France in 2016.

**Results:**

Regardless of the time of serum collection, we found remarkably high overall seropositivity (94.3–98.3%) for nAb against both EV-D68 strains. Geometric mean titres increased in an age-dependent manner.

**Conclusions:**

Our data suggest that EV-D68 has been circulating in the Netherlands for decades and that the enterovirus surveillance does not accurately capture the prevalence of this clinically relevant pathogen.

## Introduction

Enterovirus D68 (EV-D68), belonging to the *Enterovirus* D species within the *Picornaviridae* family, was first isolated in 1962 but not frequently detected before 2010 when it started causing large outbreaks of severe respiratory illness worldwide [1-5]. Clinical symptoms commonly associated with EV-D68 infection include fever, wheezing, cough and dyspnoea [1]. Young children and individuals with underlying conditions are at high risk of developing severe lower respiratory tract disease requiring admission to an intensive care unit (ICU) and mechanical ventilation [1,2,5]. The characteristics of EV-D68, such as acid lability of the virions, the respiratory transmission route and symptomatology in patients, resemble those described for the related rhinoviruses [6]. However, similar to poliovirus (PV) and enterovirus A71 (EV-A71), EV-D68 has the potential to spread to the central nervous system (CNS) causing neurological complications [2]. Acute flaccid myelitis (AFM) in children has been associated with EV-D68 infection [7-12].

Based on the viral capsid protein VP1 nucleotide sequence, EV-D68 isolates are classified into three clades A to C, all of which co-circulate globally [3]. In the Netherlands, EV-D68 has been detected sporadically since 1996 and the first upsurge of EV-D68 cases was reported in 2010 [4]. Continuous circulation has been observed from 2011 to 2016, with severe outbreaks in 2014 and 2016 [13-15]. Surveillance of enteroviruses (EV) occurs via the national public health networks in the context of the World Health Organization (WHO) polio surveillance, by detection of viruses from patients [16]. However, as most EV infections are asymptomatic or cause mild disease and since EV diagnostic testing is performed primarily on stool samples, detection rates are likely to account for only a minority of the true EV-D68 incidence [17].

Presence of neutralising antibodies (nAb) in serum is a widely accepted correlate of immunity and protection against severe disease associated with EV infection [18]. Thus, age-stratified serosurveys of nAb are a valuable method of understanding the prevalence of EV-D68 and evaluating the risk of an outbreak among the general population. As a part of the European Non-Polio Enterovirus Network (ENPEN) [19], we aimed to characterise the seroprevalence of nAb against EV-D68 among children and adults in the Netherlands. 

## Methods

We screened sera collected from the population in the Netherlands before and after the 2010 EV-D68 upsurge against two strains of EV-D68: the prototype Fermon strain so that data would be comparable to previous studies done with the Fermon strain [20] and a genotype B3 clinical isolate from 2016, a contemporary circulating strain in Europe. We analysed the seropositivity and nAb titre distribution in the context of time of collection, age, sex and virus strain.

### Enterovirus D68 viruses and cell lines

The EV-D68 Fermon prototype strain (isolated in 1962) was obtained from the National Institute for Public Health and the Environment (RIVM, Bilthoven, the Netherlands). The EV-D68 genotype B3 clinical strain was isolated from a patient in 2016 in France and was a kind gift from Dr Bailly (Université Clermont Auvergne, Clermont-Ferrand, France). Both virus strains were cultured at 37 °C, 5% CO_2_ in rhabdomyosarcoma cell line (RD99; American Type Culture Collection, Manassas, United States (US)). Cells were maintained in Eagle's minimum essential medium (EMEM; Lonza, Basel, Switzerland) supplemented with 8% heat-inactivated fetal bovine serum (FBS; Sigma-Aldrich, St. Louis, US), streptomycin (100 µg/mL; Lonza Bio Whittaker), penicillin (100 U/mL; Lonza Bio Whittaker), non-essential amino acids (NEAA; ScienCell Research Laboratories, Carlsbad, US) and L-glutamine (200 nM; Lonza, Basel, Switzerland). Chloroform treatment of the virus stocks was performed as described in the WHO Polio Manual [21]. Briefly, 10% (v/v) chloroform (Sigma-Aldrich, St. Louis, US) was added to each virus culture and vortexed vigorously for 5 min. Chloroform was removed by centrifugation for 10 min at 3000 rpm. The 50% tissue culture infective dose (TCID50) of virus stocks was determined by means of end-point dilution using the Reed and Muench method [22].

### Serum samples

We screened 280 anonymised serum samples from Dutch individuals aged 0–79 years. We used two independent sets of samples collected at time points before and after the 2010 EV-D68 upsurge in the Netherlands. Sera from 2006 and 2007 were obtained from the RIVM as part of the PIENTER2 study (Dutch acronym for the survey on the immunisation effect in the Netherlands for evaluation of the national immunisation programme: *Peiling Immunisatie Effect Nederland Ter Evaluatie van het Rijksvaccinatieprogramma* [23]). 

### Neutralisation assay

The sera were tested using a previously described neutralisation assay [24]. Heat-inactivated sera were serially diluted in 96-well microtitre plates in a volume of 50 µL per well and incubated with 100 TCID50 per 50 µL per well of EV-D68. Subsequently, 100 µL of RD99 cells were added and incubated for 7 days. Neutralising titres were calculated based on cytopathogenic effect using the Reed and Muench method and reported as the reciprocal titres of serum dilutions exhibiting 50% neutralisation [22]. An nAb titre of ≥ 1:8 was considered positive. In agreement with previous publications [20], we defined titres 8–64 as ‘low’, 64–128 as moderate, 128–512 as ‘high’ and >512 as ‘very high’.

### Statistical analysis

Data were grouped in categories based on the following: the EV-D68 virus strain used in the assay (prototype Fermon or genotype B3 clinical isolate), serum collection time point (2006–07 or 2015–16), serum donor sex (male or female) and serum donor age. The overall EV-D68 nAb seroprevalences between different groups were compared using chi-squared tests. Kruskal–Wallis test with Dunn’s post hoc analysis was used to compare the overall and the age-stratified geometric mean titres between the prototype Fermon strain and the genotype B3 clinical isolate. One-way ANOVA with Tukey’s multiple comparisons test was used to compare the geometric mean titres between the age groups. Children younger than 1 year were excluded from the overall seroprevalence and overall geometric mean titre analyses because of the potential presence of maternal antibodies against EV-D68. Data were analysed using IBM SPSS Statistics for Windows, Version 23.0 (IBM Corp., Armonk, US) and GraphPad Prism 7 (GraphPad Software Inc., La Jolla, US) with a significance level of p < 0.05.

### Ethical statement

The sera had been collected by population-based sampling approved by the Medical Ethics Testing Committee of the Foundation of Therapeutic Evaluation of Medicines (ISRCTN 20164309) [25]. Sera collected between 2015 and 2016 were residual samples from hospitalised patients and staff at the University Medical Centers (Amsterdam, the Netherlands). No ethical approval is required for anonymous use of residual serum in the Netherlands.

## Results

### High overall seropositivity for enterovirus D68 neutralising antibodies

**Figure 1:**
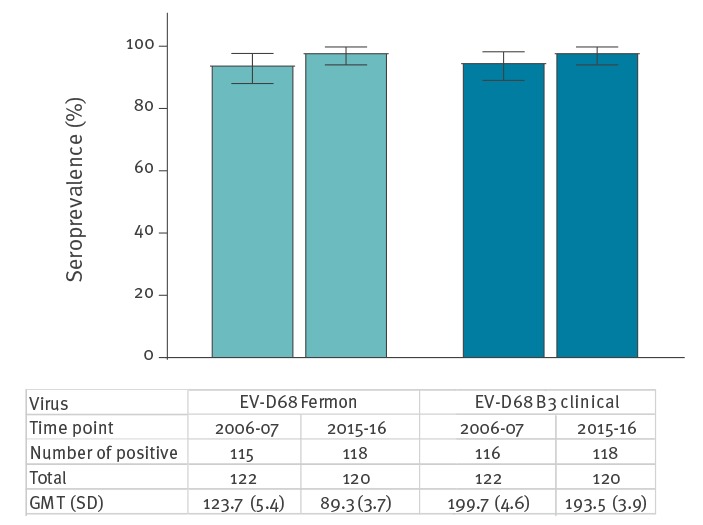
Overall seroprevalence and geometric mean titre of neutralising antibodies against enterovirus D68 (prototype Fermon strain or a genotype B3 clinical isolate), the Netherlands, 2006–07 and 2015–16 (n = 280)

EV: enterovirus; GMT: geometric mean titre; nAB: neutralising antibodies; SD: standard deviation.

As depicted in Figure 1, the overall EV-D68 nAb seroprevalence rates and geometric mean titres (GMT) were high, with no statistically significant differences between time points before and after the 2010 EV-D68 upsurge in the Netherlands or between the virus strains. The overall nAb seroprevalence in the 2006 and 2007 sera against the prototype Fermon strain was 94.3% (95% confidence interval (CI): 88.0–97.7) with a GMT of 123.7 (standard deviation (SD): 5.4) and in the 2015 and 2016 sera, it was 98.3% (95% CI: 94.0–99.8) with a GMT of 89.3 (SD: 3.7). The overall nAb seroprevalence in the 2006 and 2007 sera against the genotype B3 clinical isolate was 95.1% (95% CI: 89.0–98.2) with a GMT of 199.7 (SD: 4.6) and in the 2015 and 2016 sera, it was 98.3% (95% CI: 94.0–99.8) with a GMT of 193.5 (SD: 3.9). No differences in the nAb seropositivity rates were found between female and male cases (data not shown).

Light grey bars: Fermon strain; dark grey bars: B3 clinical isolate. Left columns: 2006–07; right columns: 2015–16. Whiskers indicate the 95% confidence intervals. Number of positives refers to subjects with titres >8.

### Age-associated increase in enterovirus D68 neutralising antibody titres

**Figure 2:**
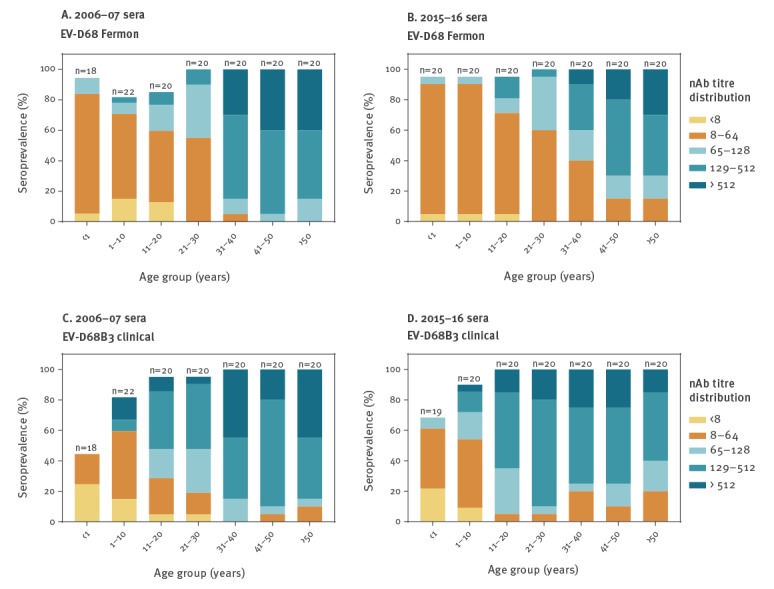
Age-stratified seroprevalence and distribution of enterovirus D68 neutralising antibodies against the prototype Fermon strain or the genotype B3 clinical isolate, the Netherlands, 2006–07 and 2015–16 (n = 280)

EV: enterovirus; nAB: neutralising antibodies.

Age-stratified analysis showed that in children below the age of 1 year the EV-D68 nAb seroprevalence was 94.4–95.0% against the Fermon EV-D68 strain and 44.4–68.4% against the genotype B3 clinical isolate (Figure 2). While more infants were seropositive for nAb against the Fermon strain than against the genotype B3 clinical isolate, their GMT values were similar against both strains (Table). Regardless of the virus strain, seroprevalence of EV-D68 nAb was 81.8–95.0% in 1–10 year-old children, while sera from adolescents and adults (age groups 11–20, 21–30, 31–40, 41–50 and above 50 years) were 85–100% positive for EV-D68 nAb (Figure 2). Children and young adults in age groups 1–10 and 11–20 years had significantly lower GMT of nAb against the Fermon strain than against the genotype B3 clinical EV-D68 isolate (Table). Most adult age groups had high GMTs against both virus strains with no statistically significant differences between the virus strains (Table). Statistical pairwise GMT comparisons between age groups indicated that children younger than 1 year and children between 1 and 10 years of age had significantly lower GMT of nAb against both the Fermon and the genotype B3 clinical isolate when compared with the GMTs in the adult age groups (adjusted p values < 0.0001; Supplementary Tables S1 and S2).

**Table:** Geometric mean titres of neutralising antibodies against enterovirus D68, serosurvey, the Netherlands, 2006–07 and 2015–16 (n = 280)

Age group (years)	Number	M/F	Mean age in years (SD)	GMT (SD) EV-D68 Fermon	GMT (SD) EV-D68 genotype B3	Adjusted p value
2006 and 2007 sera^a^						
< 1	18	9/9	0.5 (0.3)	18.2 (2.6)	10.5 (3.2)	> 0.9999
1–10	22	11/11	5.2 (3.1)	19.6 (3.1)	55.6 (7.4)	**0.0241**
11–20	20	10/10	15.6 (3.0)	32.0 (3.8)	130.3 (3.7)	**0.0013**
21–30	20	10/10	25.7 (3.0)	66.3 (1.9)	160.3 (3.6)	0.1263
31–40	20	10/10	35.7 (3.0)	369.6 (2.5)	453.8 (2.7)	> 0.9999
41–50	20	10/10	45.8 (3.0)	530.1 (2.4)	304.4 (2.4)	0.9507
> 50	20	10/10	65.8 (8.9)	530.1 (2.7)	449.4 (3.2)	> 0.9999
2015 and 2016 sera^b^						
< 1	20	10/10	0.5 (0.3)	24.7 (2.0)	13.6 (3.1)	0.6094
1–10	20	10/10	5.5 (3.1)	23.8 (2.2)	49.3 (4.7)	0.2558
11–20	20	10/10	16.1 (2.9)	43.8 (2.8)	219.2 (3.7)	**< 0.0001**
21–30	20	10/10	26.1 (3.0)	66.3 (1.7)	339.4 (2.0)	**< 0.0001**
31–40	20	10/10	35.9 (3.0)	121.5 (2.8)	265.2 (3.3)	0.1742
41–50	20	10/10	46.0 (3.0)	230.7 (3.8)	301.7 (3.2)	> 0.9999
> 50	20	10/10	64.2 (8.2)	260.7 (3.2)	178.3 (3.6)	> 0.9999

EV: enterovirus; GMT: geometric mean titre; M/F: male/female; SD: standard deviation.

^a^ Population-based sampling, National Institute for Public Health and the Environment (RIVM), Bilthoven.

^b^ Residual sera from hospitalised patients and staff, Academic Medical Center, Amsterdam.

Numbers in bold indicate statistical significance.

### Enterovirus D68 clinical surveillance in the Netherlands, 1996–2017

**Figure 3:**
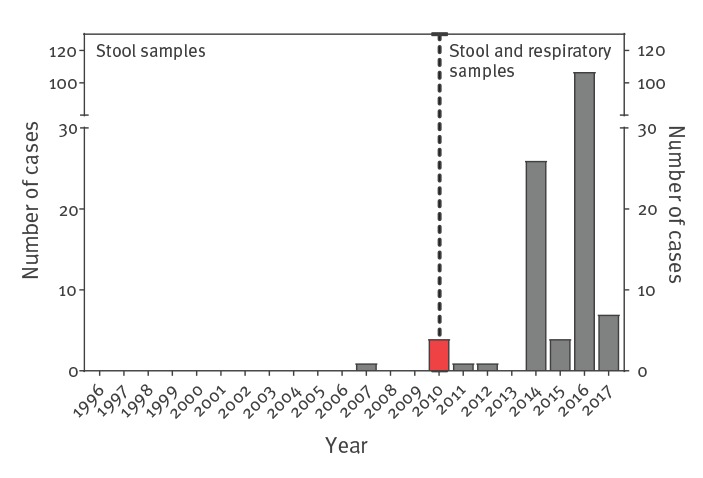
Clinical enterovirus surveillance data on enterovirus D68, the Netherlands, 1996–2017 (n = 151)

We extracted the EV-D68 case numbers reported during 1996 to 2017 in the Netherlands from the national Clinical Enterovirus Surveillance (CEVS) database (Figure 3) [26]. From 1996 to 2010, enterovirus testing was performed primarily on stool samples and few cases were observed. Because the 2010 EV-D68 outbreak was discovered via primary care surveillance done by Nivel, the Dutch Institute for Health Care Research, in respiratory samples that were not included in the CEVS, this outbreak is not visible in Figure 3 [4]. EV-D68 testing in respiratory samples has been gradually implemented following the 2010 outbreak. After 2010, 146 cases have been confirmed, most of them during an outbreak in 2016 [13].

From 1996 to 2010, enterovirus testing was performed primarily on stool samples, showing low numbers of cases in 2010, the first outbreak year in the Netherlands (highlighted in red). Since 2010, also respiratory samples have been increasingly tested for EV-D68.

## Discussion

EV-D68 was only sporadically detected before 2010 when it suddenly caused large outbreaks of severe lower respiratory infections and polio-like illness worldwide, but particularly in North America [1,4,5,9]. Concerns were raised that EV-D68 was developing from an infrequent cause of mild disease to a major human pathogen with neurovirulent properties [1]. This study is the first serological investigation into the prevalence of EV-D68 among the Dutch population.

In line with previous sero-epidemiological studies from Finland and China, with seroprevalence rates from 90 to 100% [20,27], the overall nAb prevalence was remarkably high in sera collected both before and after the first reported EV-D68 upsurge in the Netherlands in 2010. The nAb were specific to both the prototype Fermon EV-D68 strain and a recent genotype B3 clinical isolate from France. Age-stratified analyses indicated that the overall EV-D68 nAb seroprevalence was approaching 90% or more already in 1–10 year-old children. The higher GMT in the older age groups is most likely explained by frequent boostering. Our data suggest that EV-D68 circulation has been endemic in the Netherlands for decades.

Antigenic drift has been proposed as a mechanism to explain the sudden EV-D68 emergence [4,28,29]. We found that Dutch children and young adults had higher nAb titres against the recent genotype B3 clinical isolate EV-D68 isolate than against the prototype strain. However, overall the sera from all time points and age groups could efficiently neutralise both EV-D68 strains with minimal differences between GMT. As we used anonymous serum collections, we were unable to relate the exposure histories of EV-D68 sample donors to our seroprevalence data. This is a limitation of our study. Cross-neutralisation by nAb elicited against other prevalent enteroviruses may be a confounding factor in our study. However, evidence of cross-neutralisation among different enterovirus serotypes is scarce [18,30]. Previously it was reported that EV-D68 could not be neutralised with the reference EV-D70 antiserum [29]. In the same report, it was suggested that a small antigenic variation between the 2014 outbreak viruses and the Fermon strain could explain differences in neutralisation titres.

We hypothesise that EV-D68 incidence in the Netherlands is underestimated based on the following: (i) in general, the majority of enterovirus infections are not reported as most infections are subclinical or cause only mild illness in healthy individuals [18]; (ii) standard molecular diagnostics cannot distinguish between rhinovirus and EV infection, and EV type-specific testing is predominantly based on stool sampling since EV are not perceived as relevant respiratory pathogens [17]; (iii) as reported previously in other countries [20,27], we observed a nearly universal prevalence of EV-D68 neutralising antibodies among the Dutch general population.

## Conclusion

We report a high level of population immunity against EV-D68 and conclude that EV-D68 has been endemically circulating in the Netherlands for decades. Our results suggest that the current EV surveillance does not accurately capture the EV-D68 prevalence in the Netherlands. In order to fully understand the EV-D68 disease burden, we propose monitoring and routine EV-D68 testing of nasopharyngeal aspirate or throat swab specimens for patients with acute respiratory presentations. Further research on antigenic variation and pathogenicity of the emerging EV-D68 variants is necessary to elucidate the factors underlying disease severity and outbreak dynamics.

## Supplementary Data

53000 Supplement
