# Supplementary material for: Enterovirus D68 serosurvey: evidence for endemic circulation in the Netherlands, 2006 to 2016
Source: Euro Surveill. 2019 Aug 29;24(35):1800671. doi: 10.2807/1560-7917.ES.2019.24.35.1800671 (PMC6724466; doi:10.2807/1560-7917.ES.2019.24.35.1800671)
Supplement: Supplement [file 1800671_WOLTHERS_supplement.pdf]

Supplementary material

This supplementary material is hosted by Eurosurveillance as supporting information alongside the article "Enterovirus D68 serosurvey: evidence for endemic circulation in the Netherlands" on behalf of the authors who remain responsible for the accuracy and appropriateness of the content. The same standards for ethics, copyright, attributions and permissions as for the article apply. Eurosurveillance is not responsible for the maintenance of any links or email addresses provided therein.

Supplementary table 1.
Age-stratified geometric mean titer comparison of neutralising antibodies against the prototype Fermon EV-D68 strain. Adjusted p values from pairwise age category comparisons. Boldface indicates a significant result.

Age categories (years)	<1	1 to 10	11 to 20	21 to 30	31 to 40	41 to 50
<1	X					
1 to 10	>0.9999	X				
11 to 20	0.1952	0.1814	X			
21 to 30	**<0.0001**	**<0.0001**	0.1608	X		
31 to 40	**<0.0001**	**<0.0001**	**<0.0001**	**<0.0001**	X	
41 to 50	**<0.0001**	**<0.0001**	**<0.0001**	**<0.0001**	0.3032	X
>50	**<0.0001**	**<0.0001**	**<0.0001**	**<0.0001**	0.1785	>0.9999

Supplementary table 2.
Age-stratified geometric mean titer comparison of neutralising antibodies against the genotype B3 clinical EV-D68 isolate. Adjusted p values from pairwise age category comparisons. Boldface indicates a significant result.

Age categories (years)	<1	1 to 10	11 to 20	21 to 30	31 to 40	41 to 50
<1	X					
1 to 10	**<0.0001**	X				
11 to 20	**<0.0001**	0.0009	X			
21 to 30	**<0.0001**	**<0.0001**	0.9193	X		
31 to 40	**<0.0001**	**<0.0001**	0.1579	0.8079	X	
41 to 50	**<0.0001**	**<0.0001**	0.39	0.9698	0.9992	X
>50	**<0.0001**	**<0.0001**	0.5463	0.9938	0.9919	>0.9999
